# Anti-plasmodial effect of plant extracts from *Picrolemma huberi* and *Picramnia latifolia*

**DOI:** 10.1186/s12936-018-2301-x

**Published:** 2018-04-04

**Authors:** Wendy Berthi, Alexa González, Alexandra Rios, Silvia Blair, Álvaro Cogollo, Adriana Pabón

**Affiliations:** 1 Malaria Group, Faculty of Medicine, Universidad de Antioquia (UdeA), Sede de Investigación Universitaria (SIU), Medellín, Colombia; 2 Jardín Botánico Joaquín Antonio Uribe, Medellín, 050010 Colombia

**Keywords:** *Picramnia latifolia*, *Picrolemma huberi*, Plant extracts, *Plasmodium falciparum*, Antimalarials

## Abstract

**Background:**

Malaria is an infectious disease caused by parasites of the genus Plasmodium, of which *Plasmodium vivax* and *Plasmodium falciparum* are the major species that cause the disease in humans. As there are relatively few alternatives for malaria treatment, it is necessary to search for new chemotherapeutic options. Colombia possesses a great diversity of plants, which are potential sources of new compounds of medical interest. Thus, in this study the antiplasmodial effect of extracts from two species of plants from the families Simaroubaceae and Picramniaceae (*Picramnia latifolia* and *Picrolemma huberi*) was evaluated in vitro and in vivo. These plants were chosen because they contain secondary metabolites with interesting medicinal effects.

**Results:**

The ethanolic extracts of both species were highly active with IC_50_: 1.2 ± 0.19 µg/mL for *P. latifolia* and IC_50_: 0.05 ± 0.005 µg/mL for *P. huberi*. The *P. latifolia* extract had a stage specific effect on trophozoites and inhibited parasite growth in vivo by 52.1 ± 3.4%, evaluated at 1000 mg/kg in Balb/c mice infected with *Plasmodium berghei*. On the other hand, evaluated at 150 mg/kg body weight in the same murine model, the ethanolic extract from *P. huberi* had an antiplasmodial effect in all the asexual intraerythrocytic stages of *P. falciparum* FCR3 and inhibited the parasitic growth in 93 ± 32.9%.

**Conclusions:**

This is the first report of anti-malarial activity for these two species of plants. Thus, *P. latifolia* and *P. huberi* are potential candidates for the development of new drugs for treating malaria.

## Background

Malaria is a worldwide public health problem; it is estimated that in the year 2015 malaria caused about 438,000 deaths, mainly in African children [1]. This disease is widely distributed in Colombia, a country with climatic, geographical and epidemiological conditions suitable for transmission. The species *Plasmodium falciparum* and *Plasmodium. vivax* are the most prevalent, with *P. falciparum* being the species that causes the most severe form of the disease [2]. As an aggravating factor in this epidemiological situation, *P. falciparum* has developed resistance to some anti-malarials, such as chloroquine, mefloquine, sulfadoxine/pyrimethamine and some artemisinin derivatives [3–12].

Therefore, it is necessary to search for new therapeutic options for the treatment of the disease. In this sense, traditional medicine provides us with numerous sources of new active compounds [13, 14]. In Latin America, plant species with medicinal properties belonging to the families* Simaroubaceae* and* Picramniaceae* have been researched. Thus, a review of plant species belonging to these families with reported medicinal activity and easy access for their collection was performed. As a result, two species were selected for evaluation because they contain secondary metabolites with interesting medicinal effects: *Picrolemma huberi* and *Picramnia latifolia*. To do so, in vitro assays in *P. falciparum* strains resistant and sensitive to chloroquine and in vivo assays in mice infected with *Plasmodium berghei* were performed.

## Methods

### Plant material

The compound leaves and stems of the *P. huberi* and *P. latifolia* plants were collected at the Joaquín Antonio Uribe botanical garden in Medellín, Antioquia, Colombia. A botanical sample of the evaluated species is deposited in JAUM (Herbarium of the Botanical Garden of Medellín), *P. huberi* under the code Tobon Juan Pablo 2392 and *P. latifolia* under the code 65508. The plant material was dried at room temperature for 7 days. The raw extracts were prepared by grinding the plant material in an industrial mill with 5 mm particles, obtaining 255.29 g of compound leaves and 135.59 g of stems from *P. huberi*, and 125.66 g of stems and 166.93 g of compound leaves from *P. latifolia*.

### Preparation of plant extracts

The extractions were carried out following the protocols previously proposed [15, 16]. Briefly, the ground plant material was sectioned; *P. huberi* was extracted by percolation of leaves/petiole/rachis, leaves, petiole/rachis and cortex using solvents of different polarity (ethanol and hexane). *P. latifolia* was extracted by percolation of bark/leaves and bark/petiole using ethanol; each percolation was performed for 72 h, with drying intervals of 24 h between each solvent. Subsequently, each extract was filtered with Whatman paper and concentrated to dryness under vacuum using the Heidolph Laborota 4001/g3 rotary evaporator (Sigma-Aldrich) [15, 16].

A second collection of *Picrolemma huberi* plant material was made, found on the Guada road, at the Guada estate (1662 meters above sea level) in the town of Amalfi, Antioquia, Colombia (coordinates of the place: 6°52′006″ N 75°8′49,9″). The plant material (Cortex) was subjected to the same extraction procedure described above [15, 16].

Finally, a 10 mg/mL stock solution was prepared from each of the individual plant extractions by diluting them in 100% dimethyl sulfoxide (DMSO). These stock solutions were then used in the subsequent biological evaluations.

### *Plasmodium falciparum* culture

Two strains with different resistance phenotypes of *P. falciparum* [17], strain 3D7 and strain FCR3 were used. For both strains, the parasite cultures were incubated at 37 °C in RPMI 1640 culture medium supplemented with 10% inactivated fetal bovine serum, 25 mM Hepes, 0.2 mM Hypoxanthine, 5% Sodium Bicarbonate, and 25 μg/mL of gentamicin sulfate (G1397 SIGMA) in the presence of 5% CO_2_, 5% O_2_ and 90% N_2_ [18].

### In vitro antiplasmodial activity assay

Several concentrations of each extract (from 100 to 0.02 µg/mL and DMSO: 0.99% for the highest concentration) were evaluated in the 3D7 and FCR3 strains; diphosphate salt of chloroquine (≥ 98%, SIGMA C6628), evaluated in a range of 1.03 μg/mL to 0.00118 μg/mL, was used as a treatment control in each assay [14]. Culture medium in the absence of the extract was used as a growth control. A suspension of parasitized red blood cells with a haematocrit of 2%, a total parasitaemia of 1%, and with a predominance > 80% of young trophozoite stage, were prepared. The cultures with the treatments were incubated at 37 °C for 48 h in the presence of 5% CO_2_, 5% O_2_ and 90% N_2_ [14]. After 48 h of incubation, 50 µL of 0.4% SYBR Green was added to each well and incubated for 10 min, to intercalate the fluorochrome into the DNA of the parasites [19]; after 10 min incubation, fluorescence emission detection was performed using the BD AccuriTM C6 flow cytometer, measured at 485 nm excitation and 530 nm emission [19]. Each concentration was evaluated in duplicate, and 2 or more independent assays were performed. To find the inhibitory concentration 50% (IC_50_), the percentages of parasitaemia were analyzed using a non-linear slope-dependent regression with GraphPad Prism™ version 5.01. An extract was considered highly active with an IC_50_ < 5 μg/mL, promising extracts with an IC_50_ of 6–15 μg/mL, extracts with moderate activity with a IC_50_ of 16–30 μg/mL, extracts with low activity with an IC_50_ of 31–50 μg/mL, and inactive extracts if the IC_50_ was > 50 μg/mL [20].

### In vitro cytotoxicity assay in the HepG2 cell line

HepG2 cells (ATCC HB-8065™) incubated at 37 °C with 5% CO_2_ in Dulbecco’s Modified Eagle Medium (DMEM) supplemented with 10% inactivated FBS, 2 nM l-glutamine and 1× Pen/strep amphotericin B were used [21]. 200,000 cells/mL were seeded in 96-well flat bottom plates (COSTAR 3599) (20,000 cells/well) [22, 23]. Seven serial concentrations of each extract (from 200 to 0.15 µg/mL in DMSO: 1.98% for the highest concentration) were evaluated in duplicate in two independent assays. For each assay, cells cultured under the same conditions in the absence of the extract were used as a growth control. FCCP (carbonyl cyanide 4-(trifluoromethoxy) phenylhydrazone) was used as a cytotoxicity control.

The cell viability reading was performed following the enzymatic micro-method MTT (3-(4,5-dimethylthiazol-2-yl)-2,5-diphenyltetrazolium bromide) described by Mosmann in 1983 [24]. Production of formazan was measured on a Multiskan Spectrum Thermo scientific spectrophotometer, measured at 570 nm excitation and 595 nm emission. The data obtained were analysed by non-linear regression with the GraphPad Prism™ version 5.01 program, to determine the cytotoxic concentration of each extract that reduced cell viability by 50% (CC_50_). An extract was considered very toxic with CC_50_ < 10 µg/mL, moderately toxic with CC_50_ 11–30 µg/mL, slightly toxic with CC_50_ 31–50 µg/mL, and potentially non-toxic with CC_50_ > 50 µg/mL [21].

### In vitro cytotoxicity assay in human erythrocytes

The haemolytic effect of the extracts was evaluated in human A+ erythrocytes at a concentration of 40% in isotonic sodium phosphate buffer (10 mM, pH 7.4) [25]. Seven serial concentrations starting at 10 µg/mL for *P. latifolia* and 0.5 µg/mL for *P. huberi* were evaluated in two independent trials. Erythrocytes treated with isotonic sodium phosphate buffer solution were used as a baseline haemolysis control, and 1% Tween 20 was used as the maximum haemolysis control. The erythrocyte suspension with the treatments was incubated at 37 °C for 1 h and the amount of haemoglobin released was determined on a Multiskan Spectrum Thermo scientific spectrophotometer at 540 nm [25]. The percentage of haemolysis was calculated with the following formula: % haemolysis = (treatment of each extract in isotonic solution)/(treatment in tween − isotonic solution) × (100) [25].

### Specific stage evaluation of extracts of *P. latifolia* and *P. huberi* in *P. falciparum*

The FCR3 strain parasite cultures were synchronized in the ring stage by lysis with 5% d-sorbitol (Sigma-Aldrich), with a haematocrit of 2% and a parasitaemia of 1% [26]. For the determination of the anti-plasmodial activity, each of the extracts was evaluated during several maturation periods, every 4 h, and in triplicate [26]. The parasite solution (200 µL) was added to Flat-bottomed 96 well plates (COSTAR 3599). A dilution of the ethanolic extracts (50 µL) were added at time 0 in the first three columns. The dishes were gently shaken and incubated at 37 °C in the presence of 5% CO_2_, 5% O_2_ and 90% N_2_. After the incubation period (4 h), the supernatant from the first three columns was removed and the parasitized erythrocytes from these wells were washed with RPMI-1640 medium; the whole dish was then centrifuged at 839 g for 10 min and 200 µL RPMI-1640 medium was added to each well of the first three columns. After this, the dilutions of the ethanolic extracts were placed in the remaining three columns and the dish was incubated for 4 more hours; after this time, the wells were washed with RPMI-1640 medium and this process was repeated every 4 h until the maturation period (of 48 h or more per cycle) was completed. After 48 h of incubation, 50 µL of 0.4% SYBR Green was added to each well and incubated for 10 min to intercalate the fluorochrome into the parasitic DNA and to detect the fluorescence emission [19]. After the 10 min incubation, fluorescence emission detection was performed using the BD AccuriTM C6 flow cytometer, measured at 485 nm excitation and 530 nm emission [19]. Finally, the data obtained were analysed by non-linear regression using the GraphPad Prism™ software version 5.01, to find the inhibitory concentration 50% (IC_50_) [21].

### Test of anti-malarial activity and toxicity in vivo

#### Ethical considerations for in vivo assays

BALB/c mice infected with the ANKA strain of *P. berghei* were used as an in vivo model for testing of the extracts. The animal protocol was approved by the ‘University of Antioquia Ethics Committee for the Experimentation of Animals’ (June 25, 2015). The animal protocol was developed as part of a project titled, “Development of new therapeutic agents for the treatment of diseases of public health importance in Antioquia (Block 1: E6 Prototype for malaria)”. The management of BALB/c mice was performed according to the regulations in The National Statute for the Protection of Animals (Law 84 of 1989) [27].

#### In vivo acute toxicity assay in BALB/c mice

To determine the toxicity of the extracts, the maximum tolerable dose (MTD) was determined [28]. To do so, a test dose of 2000 mg extract/kg body weight, of each ethanolic extract, administered by oral route (through a nasogastric tube), was evaluated; six BALB/c mice (three mice for each treatment), both males and females with 20 ± 2 g body weight were used. If at least one of the three mice died in a treatment group, the dose was decreased 3.2 times until no deaths were observed among the three mice.

All mice were euthanized at the end of treatment (day 7) or at any sign of pain or suffering from the mice (e.g. if the animals were underweight accompanied by pain when touching them, little response to stimuli, among others signs). Euthanasia was performed in a CO_2_ chamber at 20 PSI, with previous tranquilization with xylazine (5 mg/kg body weight) and anesthesia with ketamine 50 mg/kg body weight. Blood samples were collected by cardiac puncture (about 1 mL of blood). Blood counts and some blood chemistry parameters such as total bilirubin and creatinine were evaluated. Additionally, for analysis of organ pathologies, liver, spleen and kidney samples were collected and fixed in 3.7% paraformaldehyde.

#### Evaluation of the antimalarial effects of the ethanolic plant extracts in vivo

This trial was based on the 4-day parasitaemia suppression test or Peters test [29]. Male or female Balb/c mice of 20 ± 2 g body weight (five mice per treatment) were infected intraperitoneally with 5000,000 red blood cells parasitized with *P. berghei*/µL. The parasitized cells were resuspended in 0.9% saline solution and administered in a volume of 100 µL per mouse [30, 31]. The percentage of parasitaemia was calculated by microscopy, using a 100× immersion objective, counting 300 red blood cells on average per field, in 11 fields, with the purpose of quantifying the proportion of parasitized red blood cells. Subsequently, the percent inhibition of parasitaemia was determined with respect to the control without treatment (DMSO). The anti-malarial activity was classified according to Rasoanaivo et al. [32], as follows: extracts with very good or good activity = 90–100% parasitaemia inhibition, extracts with good to moderate activity = 50–90% inhibition, extracts with moderate to weak activity = 10–50% inhibition, inactive extracts = 0% inhibition. SPSS version 24.0.0 was used for statistical analysis. The mean percentages of parasitaemia obtained from the different treatments with the plant extracts were compared with their respective controls. Subsequently, the Tukey post hoc test with a significance of P < 0.05, was performed to identify if there were significant differences between the parasitaemias of the treatments with the ethanolic extracts of *P. latifolia* and *P. huberi*, with respect to untreated controls.

## Results

### In vitro antiplasmodial and cytotoxic activity of the plant extracts studied

The in vitro antiplasmodial activity of 10 extracts of *P. latifolia* and *P. huberi* plants was evaluated in FCR3 and 3D7 strains of *P. falciparum*; the results are described in detail in Table 1. Chloroquine was used as control, with an IC_50_ of 0.0057 µg/mL for the strain 3D7 (chloroquine sensitive phenotype) and IC_50_ of 0.064 µg/mL for strain FCR3 (chloroquine resistant phenotype).

**Table 1:** Antiplasmodial activity and in vitro cytotoxicity values of the ethanolic extracts from *P. latifolia* and *P. huberi*

Code	Description	Activity		Cytotoxicity	
		IC_50_ FCR3 ± SD	IC_50_ 3D7 ± SD	CC_50_ ± SD	SI
*P. latifolia*	Bark/leaves. Extraction by percolation with ethanol	7.6 ± 6.3	7.0 ± 0.83	146.6 ± 19.6	19.4
	Bark/petiole. Extraction by percolation with ethanol	1.3 ± 0.09	1.21 ± 0.19	3.5 ± 0.48	2.9
*P. huberi*	Leaves/petiole/rachis. Extraction with hexane	39.3 ± 9.3	156.9 ± 12.7	142.5 ± 31.7	1.2
	Leaves. Extraction with hexane	19.2 ± 0.2	51.8 ± 4.8	143.7 ± 36.0	4
	Petiole/rachis. Extraction with hexane	16.5 ± 0.01	19.0 ± 2.5	53.7 ± 6.7	3
	Petiole/rachis. Degreasing with hexane; extraction with ethanol/water (90:10).	0.2 ± 0.04	0.33 ± 0.08	5.8 ± 0.8	21.9
	Cortex. Extraction with hexane.	14.4 ± 0.04	17.7 ± 2.3	21.8 ± 0.3	1.4
	Cortex. Extraction with ethanol/water (90:10)	0.05 ± 0.0	0.09 ± 0.005	3.6 ± 0.3	51.4
	Cortex. Degreasing with hexane; extraction with ethanol/water (90:10).	0.04 ± 0.01	0.08 ± 0.03	0.38 ± 0.0	6.3
*P. huberi* AMALFI	Cortex. Extraction with ethanol/water (90:10)	0.18 ± 0.02	–	–	–
CQ	–	0.064 ± 0.01	0.0057 ± 0.0	73.4 ± 11.4	–
FCCP	–	–	–	0.36 ± 0.04	–

The data correspond to the inhibitory concentration 50 (IC_50_) ± standard deviation (DS), cytotoxic concentration 50 (CC_50_) ± standard deviation (SD) and calculated selectivity indexes (SI) of extracts from *P. latifolia* and *P. Huberi* Values are shown in µg/mL. Two or three independent trials were performed for each extract. Chloroquine (CQ) was used as a control for Plasmodium activity, FCCP (carbonyl cyanide 4-(trifluoromethoxy) phenylhydrazone) was used as a toxicity control

In the FCR3 strain, the extracts from *P. latifolia* species presented IC_50s_ from 1.33 to 7.57 µg/mL, and were considered as highly active extracts [20]. The IC_50s_ for *P. huberi* were from 0.04 to 39.33 µg/mL, of which the bark extract dissolved in ethanol:water (90:10) (with hexane degreasing pretreatment) and the bark extract dissolved in ethanol:water (90:10) without the pretreatment, showed high antiplasmodial activity with IC_50s_ of 0.04 and 0.05 µg/mL, respectively. The extract obtained from the *P. huberi* plant that was collected in the town of Amalfi, shows an IC_50_ of 0.18 µg/mL, maintaining the highly active extract classification.

For the 3D7 strain, inhibitory concentrations from 0.07 to 1.21 µg/mL were observed for *P. latifolia* extracts. Extracts from *P. huberi* showed a range of IC_50s_, from 0.08 to 156.9 µg/mL.

Regarding the evaluation of in vitro toxicity of *P. latifolia* extracts in HepG2 cells, the CC_50_ of the bark/petiole ethanolic extract was 3.5 µg/mL and the CC_50_ of the ethanolic extract of bark/leaves was 146.6 µg/mL. On the other hand, the toxicity classifications for *P. huberi* extracts ranged from very toxic (CC_50_ = 0.38 µg/mL) to potentially nontoxic (CC_50_ = 143.7 µg/mL) [21].

The leaves of the two species of plants tested were not toxic in HepG2 cells. The CC_50_ of the *P. latifolia* bark/leaves ethanolic extract was 146.6 µg/mL. Similarly, the CC_50_ of the hexane extract from *P. huberi* leaves/petiole/rachis and the hexanic acid extract from the *P. huberi* leaves alone, was 142.5 and 143.7 µg/mL, respectively. In contrast, some degree of toxicity was observed in the extracts obtained from bark, petiole and rachis, where no leaves were present, from both plant species regardless of the solvent used; with CC_50s_ < 50 µg/mL (slightly toxic extracts, moderately toxic and very toxic).

The selectivity index (SI) of extracts of *P. latifolia* and *P. huberi* were assessed and demonstrate specific antiplasmodial activity rather than toxicity in HepG2 cells, since most of the indices were ≥ 2. The extracts with an SI < 2, such as the hexane extract from bark, would not be good candidates for the treatment of malaria. Overall, the hexane extracts induced more toxicity when compared to the ethanolic extracts.

The haemolytic capacity of *P. latifolia* and *P. huberi* extracts was evaluated in healthy red blood cells. The ethanolic extract of *P. latifolia* evaluated at a concentration of 10 µg/mL (8.5× IC_50_) showed a haemolysis percentage of 1.35%, while the ethanolic extract of *P. huberi* evaluated at a concentration of 0.5 µg/mL (10× IC_50_) showed a haemolysis percentage of 0.43%. Thus, these ethanolic extracts do not have haemolytic capacity in healthy red blood cells when compared with the total haemolysis control (1% Tween 20). The cytotoxic activity of all the plant extracts evaluated in human erythrocytes is shown in Table 2.

**Table 2:** Hemolytic activity of extracts from *P. latifolia* and *P. huberi* plants in human erythrocytes

Sample	% hemolysis	Optical density (nm)
Tween 20 1%	100	4.28
DMSO 10%	0	0.2
Isoosmotic buffer	0	0.17
*P. huberi* (0.5 µg/mL) (10× IC_50_)^a^	0.43	0.19
*P. latifolia* (10 µg/mL) (8.5× IC_50_)^b^	1.35	0.21

The data correspond to the percentage of hemolysis in human A+ erythrocytes. Two independent assays were performed for each ethanolic extract. Measurements were made after 1 h incubation of erythrocytes with ethanolic extracts. As control for total hemolysis (100%), 1% Tween 20 was used

^a^10× IC_50_: ethanolic extract of *P. huberi* evaluated at a concentration 10 times higher than the IC_50_

^b^8.5× IC_50_: ethanolic extract of *P. latifolia* evaluated at a concentration 8.5 times greater than IC_50_

### Specific in vitro activity of extracts from *Picramnia latifolia* and *Picrolemma huberi* plants in *Plasmodium falciparum* strain FCR3

**Fig. 1:**
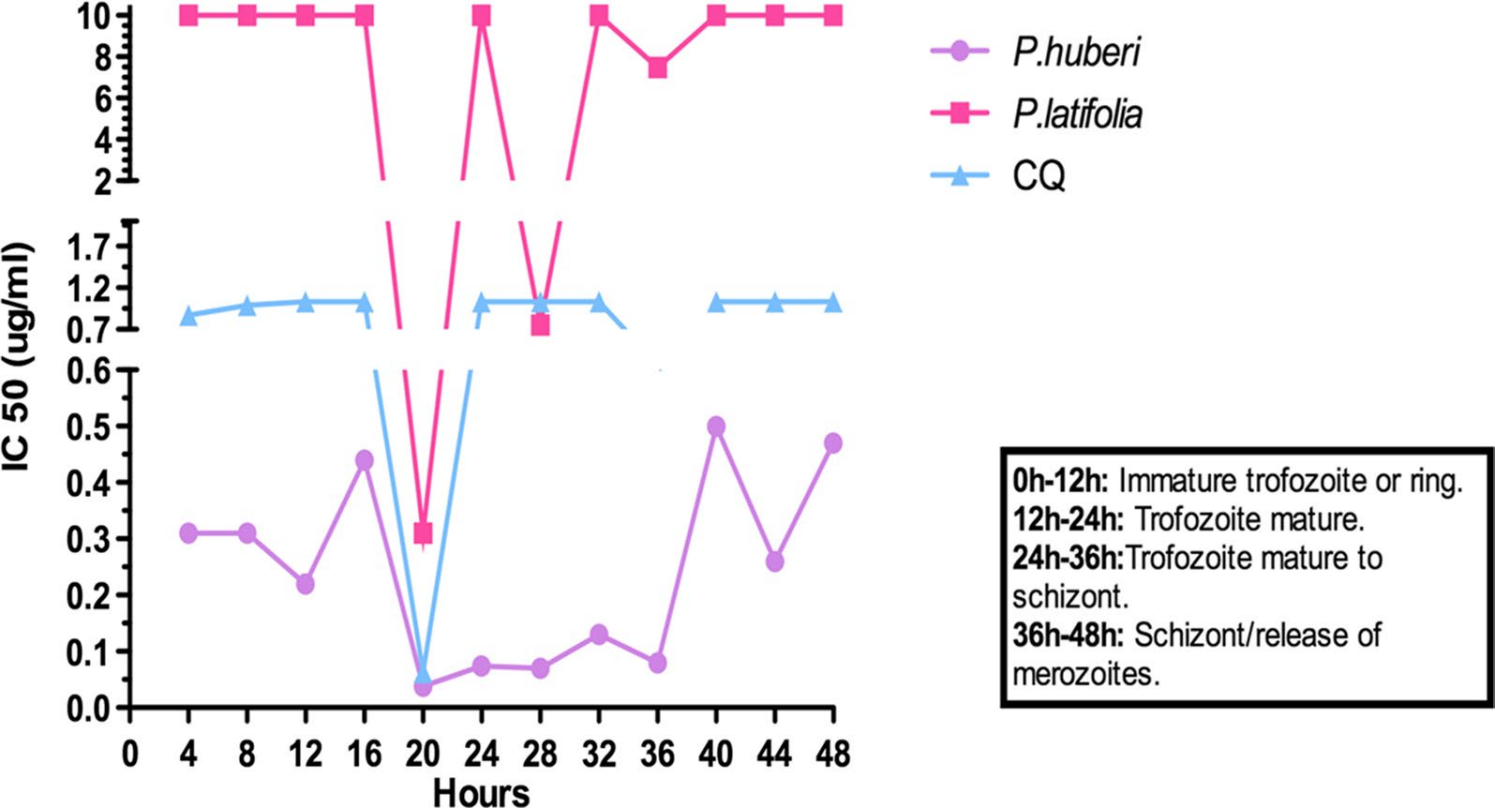
Stage specific activity of the ethanolic extracts of *P. latifolia* and *P. huberi.* The figure shows the inhibitory concentration 50 found for each ethanolic extract from 2 to 3 independent assays. Seven serial concentrations were used, for *P. latifolia* it started at 10 µg/mL and for *P. huberi* it started at 0.5 µg/mL. Measurements were made every 4 h, from 0 to 48 h, for each of the ethanolic extracts. The colors indicate the different treatments evaluated. Chloroquine (CQ) was used as a control

The concentrations of *P. latifolia* extracts that inhibited parasite growth by 50% at 20 and 28 h was 0.3 and 0.7 µg/mL, respectively. During the life cycle of *P. falciparum*, 20 and 28 h correspond to the young and mature trophozoite stage of development [32]. Growth of parasites was inhibited by 50% between 20 and 36 h when they were exposed to plant extract concentrations of 0.05–0.1 µg/mL from the *P. huberi* species. This time period (20–36 h) covers all the asexual intraerythrocytic stages of parasite development, from juvenile trophozoites to schizonts (Fig. 1).

### Evaluation of toxicity and anti-malarial activity in vivo of plant extracts from *Picramnia latifolia* and *Picrolemma huberi*

For *P. latifolia*, the MTD was established at 2000 mg/kg body weight. This indicates that the ethanolic extract from the plant was not toxic for Balb/c mice at 2000 mg/kg weight in oral treatments. For *P. huberi*, the MTD was established below 205 mg/kg body weight.

Blood samples were taken to evaluate blood chemistries and blood chemical parameters to determine possible alterations in the liver function of the 3 mice under treatment. Mild leukocytosis was found with a value of 11.3 × 10^3^/µL (reference range in mice is 5.0–8.93 × 10^3^/µL) with predominance of neutrophilia and mild thrombocytosis with a value of 672.00 × 10^3^ µL (reference range in mice: 200–590 × 10^3^ µL) in mice treated with *P. latifolia* at 2000 mg/kg (recommended maximum concentration for evaluations). Increased aspartate transaminase enzyme (AST) values were observed in the blood chemistry analysed: 17.8 μ/L, 3 times above the reference values (reference range in mice: 0.0–5.0 μ/L), indicating possible alterations in the liver function in mice treated with *P. latifolia* at 2000 mg/kg and with *P. huberi* at 61.03 mg/kg. Since the maximum tolerable concentration recommended by the 2008 OECD guidelines was used, these alterations suggest that the mice exhibited basic immunological reactions to foreign agents. The Alanine transaminase enzyme (ALT), creatinine and total bilirubin were not altered. There were no changes in animal weight or signs of pain when touched [27].

Samples of liver, kidney and spleen tissue were taken from mice treated with the MTD of the ethanolic plant extracts from *P. latifolia* and *P. huberi* for pathology studies. In mice treated with the *P. latifolia* ethanolic extract, acute splenitis or spleen inflammation was observed, characterized by an increase in organ size. A slight abnormal increase in liver size and an increase in the size of the hepatocyte nucleus or moderate to severe karyomegaly were observed. No tissue damage was observed in the kidney. In mice treated with the *P. huberi* ethanolic extract, a slight increase in hepatocyte nucleus size, binucleation, congestion, and macrophages in the liver were observed. Additionally, a slight abnormal increase in kidney size or hyperplasia, and mild congestion in the spleen were observed. Further studies are needed to identify the importance of these lesions in the functioning of the organs.

**Fig. 2:**
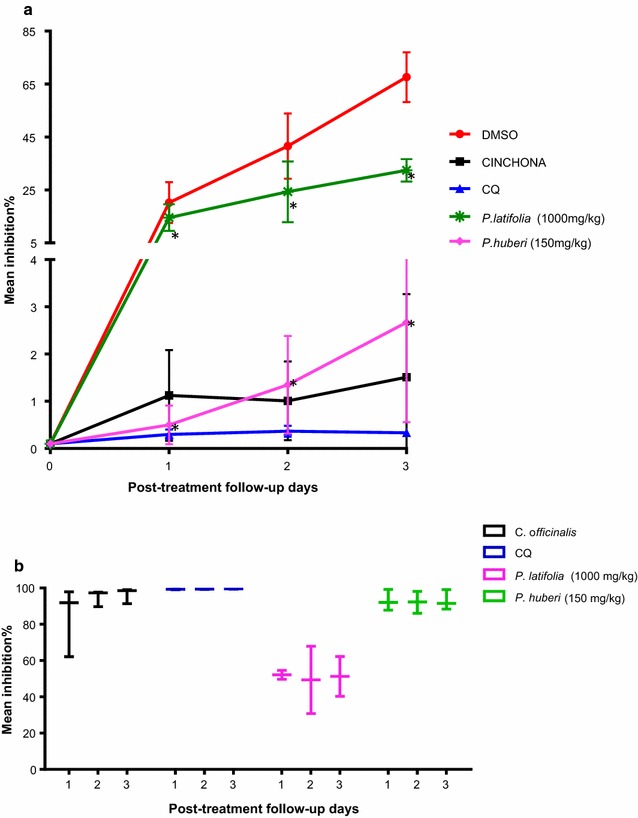
Anti-malarial activity of the ethanolic extracts from *P. latifolia* and *P. huberi*. **a** Averages of the parasitemia percentages in the mice treated with the ethanolic plant extracts. The bars indicate the SD. As control of parasitemia elimination, chloroquine (CQ) at 10 mg/kg body weight and an ethanol extract of *Cinchona officinalis* at 500 mg/kg were used. *Significant difference with DMSO of P = 0.001. **b** Averages of the percentages of inhibition of maxima and minima ± SD of mice treated with the ethanolic extracts from the evaluated plants. As control of parasitemia elimination, chloroquine (CQ) at 10 mg/kg body weight and an ethanol extract of *Cinchona officinalis* at 500 mg/kg body weight were used

Table 3 shows the anti-malarial activity of each extract using the in vivo mouse model. Parasitaemia is expressed as the mean percentage of parasitized red blood cells for each treatment group compared to the vehicle control group ± the standard deviation (± SD). Inhibition of parasitaemia is expressed as the proportion of total red blood cells analysed without parasites (mean percentage ± SD) from the mice treated with plant extracts or chloroquine. There was a near elimination of 100% in the positive parasitaemia elimination controls. For mice treated with an extract of *Cinchona officinalis* (positive control group for parasitaemia removal by a quinine-containing plant extract), parasitaemia inhibition rates of 96.3 ± 4,3% were observed, and for mice treated with chloroquine, inhibition was 99.7 ± 1.5% (Table 3, Fig. 2b).

**Table 3:** Parasitemias and averages of percent parasitemia inhibition in mice treated with the ethanolic extract of *P. latifolia* and with the ethanolic extract of *P. huberi*, during 3 days of follow-up

		Average parasitemia			Average % inhibition		
Treatment	Dose (mg/kg)	1 day POST-T ± SD	2 days POST-T ± SD	3 days POST-T ± SD	1 day POST-T ± SD	2 day POST-T ± SD	3 day POST-T ± SD
CLOROQUINE	10	0.5 ± 0.4	0.6 ± 0.5	0.5 ± 0.3	99.7 ± 1.5	99.3 ± 1.3	99.5 ± 0.8
C. *officinalis*	500	1.1 ± 1.0	1 ± 0.8	1.5 ± 1.8	84.0 ± 19.2	94.9 ± 4.5	96.3 ± 4.3
P*. latifolia*	1000	14.5 ± 5.0	24.3 ± 11.5	32.4 ± 4.2	52.1 ± 3.4	49.4 ± 26.2	51.3 ± 15.5
P. *huberi*	150	0.5 ± 0.4	1.4 ± 1.0	2.7 ± 2.1	93.0 ± 32.9	92.1 ± 42.6	93.0 ± 42.7
DMSO vehicle	–	20.2 ± 7.7	41.6 ± 12.3	67.6 ± 9.4	–	–	–

The data correspond to the mean ± standard deviation (SD) of four or five mice treated for 4 days with the ethanolic extracts of the plants *Picramnia latifolia* and *Picrolemma huberi*. To calculate the % inhibition the vehicle DMSO was used as the control; additionally, chloroquine at 10 mg/kg body weight and an ethanol extract of *Cinchona officinalis* at 500 mg/kg were used as the parasitemia elimination controls

*POST-T* post treatment

Regarding the specific treatments, parasitaemia was inhibited by 52.1 ± 3.4% (effective dose 50) in the mice treated with the 1000 mg/kg dose of *P. latifolia* ethanolic extract (Fig. 2b), and classified as an extract with good to moderate activity [33]. Statistical analysis of the means with a Tukey post hoc test revealed significant differences between the parasitaemias observed during the three-day follow-up treatment with the 1000 mg/kg dose, when compared to the DMSO vehicle control treatment (P < 0.05), (Fig. 2a).

On the other hand, in the evaluations carried out with *P. huberi*, parasitaemia inhibition averages of up to 93 ± 32,9% were determined at the 150 mg/kg dose (Table 3, Fig. 2b). Thus, according to Rasoanaivo et al. [32] the *P. huberi* extract was classified as a very good extract or one with good activity. Statistical analysis of the means with a Tukey post hoc test revealed significant differences (P < 0.05) between the parasitaemias observed during the 3 day follow-up in the mice treated with the 150 mg/kg dose of *P. huberi* extract, relative to the DMSO vehicle control group (Fig. 2a).

In the mice treated with chloroquine as a positive control for parasitaemia elimination during the anti-malarial activity experiment, the following pathologies were observed in the tissues collected: focal hepatitis, karyomegaly and binucleation in the liver; reactive hyperplasia and mild glomerular congestion in the spleen; and mild renal congestion. In the mice treated with the *C. officinalis* ethanolic extract, the following pathologies were observed: mild hepatocyte degeneration with moderate binucleations and follicular lymphoid hyperplasia in the liver; moderate lymphoid follicle hyperplasia in the white pulp and hydropic degeneration in the spleen; and slight thickening of the glomerular membranes in the kidney. As for the mice treated with vehicle control (DMSO), we observed the following pathologies in the tissues collected: non-suppurative periportal hepatitis, hydropic degeneration and fat degeneration were present with malaria pigment and parasites present in the liver; lymphoid hyperplasia with parasites present in the spleen; and turbid and vacuolar degeneration, congestion, necrosis and membranoproliferative glomerulonephritis with presence of parasites in the kidney.

The pathology results from the mice treated with the ethanolic extract of *P. latifolia* were the following: parasitic hepatitis characterized by hyperplasia, vacuolar degeneration, karyomegaly, and with presence of malaria pigment in the liver; mild acute congestive splenitis characterized by congestion with the presence of malaria pigment in the spleen; mild renal congestion characterized by vacuolar degeneration with presence of malarial pigment in the kidney; and finally, alterations in the liver enzymes alanine transaminase (ALT) and aspartate transaminase (AST) were observed. For mice treated with the ethanolic extract of *P. huberi*, the following pathologies were observed: mild vacuolar degeneration and congestion in the liver; mild reactive hyperplasia and mild apoptosis in the spleen; nephrosis and mild congestion in the kidney; and alterations in the liver enzymes ALT and AST.

## Discussion

It is necessary to explore new chemotherapeutic options for the treatment of malaria, such as molecules of natural origin that function as potent anti-malarials. The plants from the Simaroubaceae and Picramniaciae families are known sources of these molecules. Due to their chemical constitution they have been the object of many studies and consequently, numerous compounds have been isolated from them [33–41].

Other investigators have classified promising ethanolic plant extracts as highly active due to their ability to eliminate *P. falciparum* in vitro [20]. In this study, two species of plants were selected for analysis, *P. huberi* and *P. latifolia*, belonging to the families *Simaroubaceae* and *Picramniaciae*, respectively. Several studies show promising results for the antiplasmodial activity of the family *Simaroubaceae* against *P. falciparum species*. More specifically, it was found that the quasinoides simalikalactone E and D, eurycomanone, isobrucein B, among others, are the most active metabolites present in these plants [38, 39, 42–44]. This group of active metabolites is considered a taxonomic marker of this plant family, since it is the most abundant group of natural metabolites that they possess and their synthesis is almost exclusive compared to other plant families. The *P. huberi* plant has two quassinoids, isobrucein B and sergeolide [45]. Furthermore, Silva and collaborators in 2009 isolated isobrucein B and sergeolide from the *Picrolemma sprucei* plant and evaluated the antiplasmodial activity of these quassinoids in the K1 *P. falciparum* strain; they obtained IC_50s_ of 1.0–4.0 µg/L [39]. On the other hand, other investigators have reported for the Picramniaciae family, compounds such as beta-sitosterol and compounds derived from anthracene and from anthraquinones (e.g. picramniosides, mayosides and benzan-thrones) [46]. In the species *P. latifolia*, picramniosides, mayosides and benzan-thrones have been identified, of which there is no known antiplasmodial activity. In this study, the ethanolic extracts of this plant species are classified as highly active against the *P. falciparum* strains FCR3 and 3D7, being the first report of antiparasitic activity of this species.

Most of the antiplasmodial activity values obtained in both strains of *P. falciparum* are very similar for all the extracts evaluated, independent of the solvent and the part of the plant used. In this study, hexane and ethanolic extracts from both plants were evaluated in an attempt to identify the type of metabolites responsible for the antiparasitic activity. The results reported here suggest that the polar metabolites present in the ethanolic extracts are more active than the non-polar metabolites present in the hexane extracts. To identify the active metabolites of these extracts and to determine if they correspond to those reported in the literature for the families of plants to which they belong, a fractionation of the ethanolic extracts is necessary [33].

The lower IC_50_ values obtained from the ethanolic extracts, mainly in those obtained from the bark and crust/petiole of the plants, indicate a higher antiparasitic activity. These results correspond to previous reports in the literature, where authors such as Yan et al. and Silva et al. [39, 41] isolated quassinoids from members of the *Simaroubaceae* family. More specifically, from the bark of the *Picrasma quassioides* species and from the stem of the *Picrolemma sprucei* species.

On the other hand, when we evaluated the toxicity of the ethanolic plant extracts in the HepG2 cell line, according to the classification reported by Garcia-Huertas et al. [21] these extracts are very toxic and potentially non-toxic extracts of *P. latifolia*. It should be noted that the toxicity of the extracts in the HepG2 cell line was not related to the type of solvent nor to the part of the plant evaluated. The results of these evaluations show moderately toxic and highly toxic extracts (CC_50_ < 30 µg/mL) in both ethanolic and hexane extracts, indicating that the metabolites extracted with each solvent have a degree of toxicity in HepG2 cells. Despite these results, the calculated SI showed greater selectivity of the *P. latifolia* and *P. huberi* plant extracts for parasites than toxicity in the HepG2 cell line. Only 2 of the 9 extracts evaluated indicated SI values lower than 2, the *P. huberi* leaves/petiole/rachis hexane extract and the hexane extract of *P. huberi* bark. These two hexane extracts possess antiplasmodial activity but may be toxic to host cells and therefore should not be considered as anti-malarial potentials [47].

Of all the extracts from *P. latifolia* and *P. huberi* evaluated, the ethanolic extract from *P. latifolia* bark/petiole and the ethanolic extract from *P. huberi* bark showed the most promising anti-malarial characteristics; are highly active extracts against the multiresistant FCR3 and 3D7 strains and have an SI greater than 2 [20, 21, 47]. Furthermore, these promising ethanolic extracts do not have haemolytic capacity in healthy red blood cells. The erythrocyte membrane is a delicate structure that can be significantly altered by interactions with plant extracts [39, 48]. Since malaria patients may present with anaemia as a consequence of increased erythrocyte rupture, extracts considered to be promising anti-malarials should not cause haemolysis, as this would represent a risk for the patient. The ethanolic extracts of *P. latifolia* and *P. huberi* plants do not have a considerable haemolytic effect considering that the evaluations were carried out with concentrations up to 10 times higher than the IC_50s_ found for the extracts (IC_50_ for *P. latifolia *= 1.2 µg/mL; IC_50_ for *P. huberi *= 0.05 µg/mL).

On the other hand, evaluations were performed to determine the stage-specific activity of ethanolic extracts in the FCR3 *P. falciparum* strain. The evaluation of the ethanolic extract from *P. latifolia* revealed an antiplasmodial action on the trophozoite stage, in both juvenile and mature trophozoites. By acting on this phase of the life cycle, the maturation of trophozoites is inhibited, avoiding the formation of new schizonts and the continuity of the asexual life cycle of the parasite. This antiplasmodial activity is observed with chloroquine, a quinoline which acts by inhibiting the metabolism of the haem group, and thus, preventing the maturation of trophozoites and also the formation of new schizonts [48–51]. In studies conducted by Morita et al. [52] synthetic endoperoxides (N-89 and N-251) were evaluated against the FCR3 *P. falciparum* strain, observing an antiplasmodial activity specific for the trophozoite stage.

When evaluating the ethanolic extract of *P. huberi* in this study, a different behavior was observed. In this case, the antiplasmodial activity was evidenced in all stages of the asexual life cycle of the parasite. Skinner et al. [51] in 1996 reported multistage antiplasmodial activity of dihydroartemisinin in two *P. falciparum* isolates. Artemisinin and its derivatives are sesquiterpene lactones with a 1,2,4-trioxane nucleus incorporating an endoperoxide bond. The mechanism of action of these compounds is still debated, but are believed to be prodrugs that are activated by reductive cleavage of the endoperoxide ring; the resulting free radicals react with susceptible groups within a range of parasite proteins, leading to damage and cell death throughout the intra-erythrocytic cycle [53]. For most of the immature ring stage of the parasite, a small flow of these activators through different metabolic pathways may be sufficient to generate a low level of activated artemisinins. In late ring stage trophozoites, haem and Fe2+ levels are potentiated by the degradation of haemoglobin causing an effective activation of the drug [53]. It should be noted that haemoglobin is hydrolyzed within the digestive vacuole by the action of several types of proteases, including plasmepsin aspartic proteases and cysteine falciparase proteases. Others have observed that treatment with cysteine protease inhibitors leads to the accumulation of undigested haemoglobin in the digestive vacuole, blocking the development of the parasite and leading to its death [54].

*Picrolemma huberi* is a species of the family *Simaroubaceae*, which surprisingly has been the subject of many studies with respect to its chemical constitution. Numerous compounds have been isolated from this family, such as quassinoids, alkaloids, triterpenes, steroids, coumarins, anthraquinones, flavonoids and other metabolites [55–58]. Most of the studies reported for the Simaroubaceae family show antiplasmodial activity for the quassinoids extracted from these plants [35, 38–40, 42, 44], with characteristics of highly active compounds [20]. Quassinoids such as isobrucein B and sergolides isolated from *P. huberi* extracts have only been evaluated in a melanoma cell line, and in colon and lung cell lines, with sergolide having a higher cytotoxicity CC_50_ in a range of 10^−7^–10^−8^ M [45]. The quassinoids are a group of degraded triterpenes, most with a C-20 skeleton and δ-lactones. Kirby and colleagues in 1989, using the incorporation of [^3^H] isoleucine or [^3^H] hypoxanthine showed that seven quassinoids derived from plants with different chemical substitutions (aylantinone, bruceanthine, brucein B, glaucarubinone, holacanthone, chaparin and glaucarubol), inhibited the synthesis of parasitic proteins, followed by inhibition of nucleic acid synthesis in human erythrocytes infected with the K1 *P. falciparum* strain in vitro [59]. In the malaria parasite, as in eukaryotic models, the quassinoids are fast and potent inhibitors of protein synthesis. This is probably due to the effects on the parasite’s ribosome. During intraerythrocytic proliferation, the malaria parasite produces its own ribosomes, and the quassinoids bind more strongly to the parasite’s ribosomes than to the host cell’s ribosomes [59]. This is a feature that may be responsible for the selective anti-malarial action of the quassinoids.

To determine if the anti-malarial action of the ethanolic extracts of *P. latifolia* and *P. huberi* is related to some of the aforementioned mechanisms of action, it is necessary to identify both the metabolites contained in the ethanolic extracts of *P. latifolia* and *P. huberi* and the possible therapeutic targets or metabolic pathways in which they may be exerting their action. Although both extracts have characteristics of a promising extract, the results of specific stage activity shows differences in the stages of action, possibly related to differences in their metabolic constitution.

When evaluating the anti-malarial activity of the ethanolic plant extracts in Balb/c mice infected with *P. berghei*, anti-malarial activity was observed for both species of plants. For *P. latifolia*, percentages of parasitaemia inhibition up to 52.1% evaluated at a concentration of 1000 mg/kg of body weight were found, the ED50 for this plant species. These are the first reports of anti-malarial activity for the species. The pathology studies revealed parasitic hepatitis, congestive splenitis and mild renal congestion with the presence of malaria pigment in the spleen and kidney. Alterations in ALT and AST liver enzymes were also observed. These results indicate possible alterations in liver function caused by the parasitic infections, although, when compared with the untreated controls that reached parasitaemias of up to 68% with serious damage to the organs, these alterations are slight.

For *P. huberi*, a mean parasitaemia inhibition percentage of 93% was determined at a concentration of 150 mg/kg body weight. These are the first results of anti-malarial activity reported for this plant species. The pathology results from the mice treated with the ethanolic extract of *P. huberi* revealed slight alterations. Specifically, there was mild vacuolar degeneration and congestion in the liver, mild reactive hyperplasia, mild apoptosis in the spleen, and mild congestion in the kidney. Additionally, there were alterations in the ALT and AST liver enzymes. These results are similar to those obtained for parasitaemia elimination controls: mice treated with chloroquine and *C. officinalis* ethanolic extract (quinine-containing). Focal hepatitis, karyomegaly and binucleation in the liver, reactive splenic hyperplasia and mild glomerular congestion in the spleen, and mild renal congestion were observed in the chloroquine-treated mice. For mice treated with the ethanolic extract of *C. officinalis*, mild hepatocyte degeneration was observed with moderate binucleations and follicular lymphoid hyperplasia in the liver, moderate lymphoid follicle hyperplasia in the white pulp and hydropic degeneration in the spleen with mild thickening of the glomerular membranes in the kidney. Comparing these alterations with those found in untreated mice, which reached parasitaemias of up to 68% with severe damage observed in the organs, these alterations are slight.

## Conclusion

The ethanolic extract from the bark/petiole of the species *P. latifolia* has an antiplasmodial action on the trophozoite stage in vitro and a parasitaemia inhibition of 52.1% in mice treated with 1000 mg/kg effective dose 50 (ED_50_) in vivo. On the other hand, the ethanolic extract from the bark of the *P. huberi* species has antiplasmodial action on all the asexual intraerythrocytic stages of the intraerythrocytic cycle. Due to this characteristic, *P. huberi* extract may be acting on basic mechanisms needed for parasite development, shared by all stages of the asexual life cycle. This extract also exhibits anti-malarial activity up to 93% with the 150 mg/kg dose in Balb/c mice infected with *P. berghei*. These are the first reports of anti-malarial activity of these plant species.

The above results support the use of plant extracts as new molecules for the development of anti-malarials.

## Abbreviations

ALT: alanine transaminase enzyme
DMSO: dimethyl sulfoxide
CC_50_: cytotoxic concentration 50%
CH_50_: haemolytic concentration 50%
IC_50_: inhibitory inhibitory
JAUM: Herbarium of the Botanic Garden of Medellín
MTD: maximum tolerable dose
MTT: 3-(4,5-dimethylthiazol-2-yl)-2,5-diphenyltetrazolium bromide)
TLC: thin layer chromatography
SI: selectivity index
WHO: World Health Organization
